# Disparities in Mental Health Care: An Annotated Bibliography for Psychiatry Training Programs

**DOI:** 10.1007/s40596-026-02311-1

**Published:** 2026-02-25

**Authors:** Alyssa C. Smith, Danielle R. Henderson, Aimee E. Patel, Danielle K. Patterson, Rachel B. Yoder, Emily G. Holmes

**Affiliations:** Indiana University School of Medicine, Indianapolis, IN USA

**Keywords:** Mental health, Psychiatry, Disparities, Annotated bibliography

## Abstract

**Objective:**

Significant disparities in mental health care exist for patients from minoritized racial and ethnic groups, including decreased access, poorer quality of care received, diagnostic bias, and worse treatment outcomes. Education and awareness of such disparities is necessary to improve outcomes; psychiatry training programs should include education on these disparities for trainees, in line with ACGME Milestone Systems Based Practice 2 and 3, Professionalism 2, and Practice-Based Learning and Improvement 1 and 2. To improve integration of these topics into psychiatry residency training, the authors created this annotated bibliography of articles on disparities in mental health care.

**Methods:**

The authors searched PubMed for articles relevant to disparities in mental health care. Two members of the author team read each article independently, evaluating for generalizability, ability to impact future practice, quality of study design, and relative ease of understanding the text and findings.

**Results:**

The annotated bibliography contains a total of 35 articles recommended by the author team, divided into sections by topics (e.g., general psychiatry, outpatient psychiatry, inpatient psychiatry, subspeciality topics), arranged by date of publication.

Significant disparities in mental health care exist for patients from minoritized racial and ethnic groups [1]. These disparities include decreased access to mental health care [1], poorer quality of care provided [2], greater vulnerability to diagnostic bias [3, 4], and worse treatment outcomes [4]. Education and awareness are necessary to improve outcomes; psychiatry training programs should include education on these disparities for trainees. This is in line with the ACGME Milestone Systems Based Practice 2 and 3, Professionalism 2, and Practice-Based Learning and Improvement 1 and 2 [5]. These milestones emphasize trainees’ need for recognizing disparities, self-reflection of one’s own practice, critical appraisal of the literature, advocating for system improvement, and participating in patient-centered discussions [5].

To improve integration of these topics into psychiatry residency training, the authors created this annotated bibliography of articles on disparities in mental health care. The goal of this project is to build a set of resources for faculty to use when developing residency didactics to interweave education about disparities throughout the curriculum. This annotated bibliography is not exhaustive, but rather meant to be used as a starting point for integration of these topics.

## Methods

A PubMed search was performed using the search “(((Mental Health Services[MeSH Terms]) OR (“Psychiatry”[Mesh])) AND (((“Ethnicity”[Mesh]) OR “Racial Groups”[Mesh]) OR “ethnology” [Subheading])) AND ((((“Health Inequities”[Mesh]) OR “Health Status Disparities”[Mesh]) OR “Healthcare Disparities”[Mesh])).” Titles and abstracts were screened by AS, and articles that did not address disparities in the provision of mental health services were excluded.

Next, full articles were read for determination of inclusion in the scoring process by AS. Additional articles were excluded if they were not relevant, if they were not review or research articles, if they had small sample sizes, or if they were included in subsequent systematic reviews. The list of articles to be excluded from scoring was reviewed by EH. Articles were then organized based on subspecialty areas of psychiatry.

The research team included doctoral-level mental health professionals with experience in graduate medical education (psychology internship and psychiatry residency and fellowships). The authors spearhead diversity, equity, and inclusivity initiatives within their respective roles, and represent expertise within a variety of treatment settings reflective of the articles collected. Authors were assigned articles based on their own areas of expertise.

For scoring, each article was assigned to two different authors, who each read the article individually and scored the article on generalizability (i.e., could these findings apply across the USA or they speak only to a very specific population), ability to impact future practice (i.e., is there a useful teaching point that can be taken from this study), quality of the study (i.e., if a review article, strength of search criteria; if limitations were well thought out and explained; strength of statistical analyses used, if any), and relative ease of understanding the text and findings. The research team met three times to discuss, refine, and reach consensus on these criteria to strive toward consistent scoring of articles. Scores for each field ranged from 1 to 3, with 3 indicating the highest quality within these categories. Total scores were compiled. Inter-rater reliability was calculated with a two-way random effects model for consistency in SPSS Version 31 (IBM, Armock, NY).

The author pairs assigned met to discuss their ratings, any disparate scores, and recommendations for inclusion in the annotated bibliography. The authors then met as a full group to discuss final recommendations. Articles were selected against other articles within the subspecialty category; therefore, the articles selected were deemed to be the strongest available for that specific topic. For selected articles older than 10 years at time of writing, a separate literature search was conducted for similar, more recent articles.

## Results

**Fig. 1:**
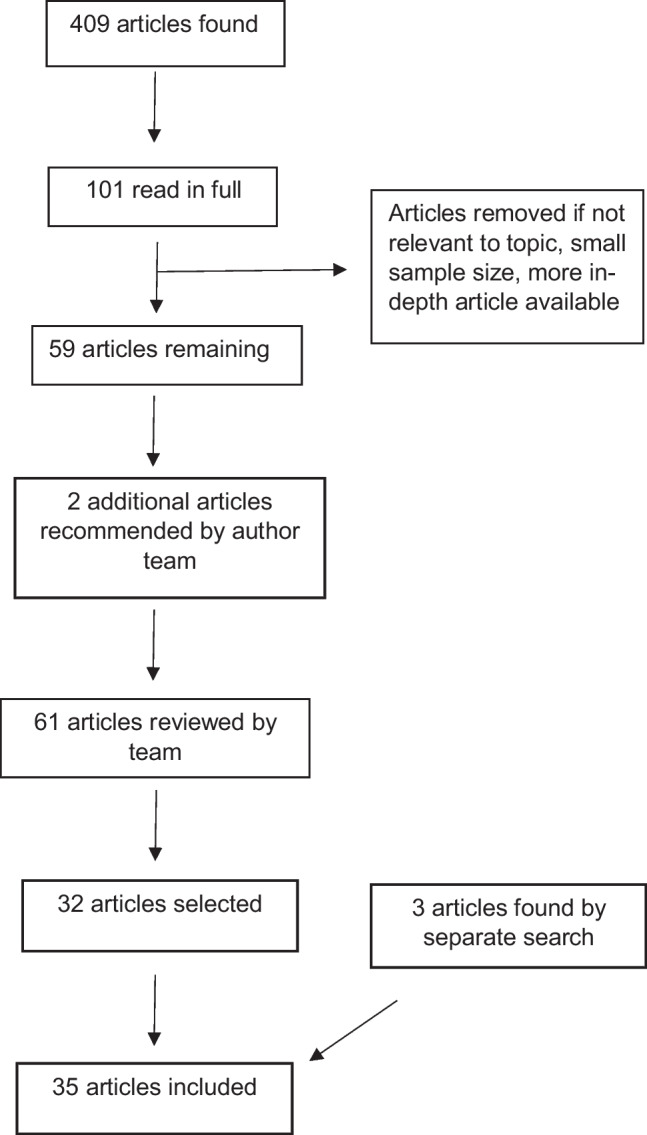
Flow chart

A total of 409 articles were found from the initial search. Of these, 101 articles were thought to be relevant to racial and ethnic disparities in the provision of mental health services based on title and abstract. These 101 articles were read in full by AS. Of these, 10 did not pertain to racial and ethnic disparities in mental health and were removed. A total of 30 more articles were removed, primarily due to a more in-depth article (e.g., systematic review or meta-analysis) also being found on the subject. Letters to the editor, studies with small sample sizes (*n* < 30), and articles that were not research studies or reviews were also excluded. This process left 59 articles. Two additional articles were recommended by the author team for review based on previous knowledge of the article, leaving 61 total articles for review. This process is shown in Fig. 1.

For the 61 articles scored by the authors, the intraclass correlation coefficient (ICC) based on a two-way random model was calculated at 0.69, indicating a moderate amount of agreement. The annotated bibliography includes the 32 articles recommended by the author team, divided into sections by topics (e.g., general psychiatry, outpatient psychiatry, inpatient psychiatry, subspeciality topics), as well as three more recent similar articles, for a total of 35 articles. Articles are arranged by date of publication. In each annotation, the authors used terminology from the annotated article with regard to race and ethnicity (minoritized/minority; Black/African American; etc.). Each annotation begins with a description of the article, followed by pertinent findings related to disparities, and concluding with the primary teaching point proposed by the bibliography authors.

### General

*Rost K, Hsieh YP, Xu S, Menachemi N, Young AS. Potential disparities in the Management of Schizophrenia in the United States. Psychiatr Serv. Jun 2011;62(6):613–618.* 10.1176/ps.62.6.pss6206_0613

This paper is a retrospective analysis of outpatient visits for individuals diagnosed with schizophrenia between 1999 and 2007. Assessment of multiple sociodemographic variables showed non-Hispanic Black patients had greater odds of hospitalization and antipsychotic medication management. Lack of insurance decreased the likelihood of hospitalization for all patients. Potential explanations for these findings are discussed and may encourage physicians to reflect on the impact sociodemographic variables may have in medication management and decisions to pursue hospitalization.

*Cook BL, Liu Z, Lessios AS, Loder S, McGuire T. The Costs and Benefits of Reducing Racial-Ethnic Disparities in Mental Health Care. Psychiatr Serv. Apr 1 2015;66(4):389–396.* 10.1176/appi.ps.201400070

This retrospective study examined the net savings of eliminating disparities in mental health care access and psychotropic drug use through identifying disparities, identifying offset, and estimating total savings. The authors found that Black and Latino patients had lower general medical expenditures, greater emergency psychiatric department expenditures, and a smaller proportion of reported outpatient mental health care. In addition, Black patients had a smaller proportion of medication use. Elimination of disparities resulted in substantially more patients receiving care and reduction of health care costs, emphasizing the costly significance of disparities.

*Shao Z, Richie WD, Bailey RK. Racial and Ethnic Disparity in Major Depressive Disorder. J Racial Ethn Health Disparities. Dec 2016;3(4):692–705.* 10.1007/s40615-015-0188-6

This literature review examined factors contributing to disparities in identifying major depressive disorder (MDD) (e.g., disparity in diagnosis of MDD, disparity in depression screening, and disparity in assessment of depressive symptoms) and disparities in depression treatment (e.g., disparities in treatment access/utilization referral, disparity in treatment outcome). African-Americans, Hispanics, and Asian Americans are less likely to receive a depression diagnosis and adequate care for depression in comparison to non-Hispanic Whites. The authors explore contributors to these disparities and give guidelines for addressing them.

*Cook BL, Hou SS, Lee-Tauler SY, Progovac AM, Samson F, Sanchez MJ. A Review of Mental Health and Mental Health Care Disparities Research: 2011–2014. Med Care Res Rev. Dec 2019;76(6):683–710.* 10.1177/1077558718780592

This review examined research on mental health care disparities funded by the NIMH. Despite increasing research in this area, disparities continue to persist. Future studies need to identify determinants of mental health and disparities at the community and policy level as well as the impact of trauma, violence, institutional discrimination, and unemployment. This study reminds psychiatrists of how entrenched such disparities are, and that we must be mindful of these when providing care to our patients.

*Pedersen SL, Lindstrom R, Powe PM, Louie K, Escobar-Viera C. Lack of Representation in Psychiatric Research: A Data-Driven Example From Scientific Articles Published in 2019 and 2020 in the American Journal of Psychiatry. Am J Psychiatry. May 2022;179(5):388–392.* 10.1176/appi.ajp.21070758

In this review, scientific articles published in the *American Journal of Psychiatry* in 2019 and 2020 were analyzed to characterize representation across racial, ethnic, gender, and sexual identities. The study found racial and ethnic underrepresentation compared to the US census. The authors provide suggestions for how investigators can improve inclusion of minoritized populations; language used when reporting race or ethnicity; assessment and reporting of sexual and gender identities; and interpretation of results and study limitations. Furthermore, psychiatrists must be mindful of disparities when reviewing research.

*Conrad JA. A Black and White History of Psychiatry in the United States. J Med Humanit. Jun 2022;43(2):247–266.* 10.1007/s10912-020-09650-6

This paper provides the history of psychiatric care from the mid-nineteenth century to the mid-twentieth century for Black and White populations to understand how our current practice is a continuation of past treatment, not a change from it. Practices that continue today include centering White culture as the norm to which everything else is measured, promoting ideas about a biological inferiority of Black people, and the use of psychiatric diagnoses to justify racial prejudices. The author asserts that we must examine our history and deepen our understanding of our current beliefs, actions, and attitudes to improve disparities.

*Ventura AMB, Hayes RD, Fonseca de Freitas D. Ethnic Disparities in Clozapine Prescription for Service-Users with Schizophrenia-Spectrum Disorders: A Systematic Review. Psychol Med. Sep 2022;52(12):2212–2223.* 10.1017/s0033291722001878

This systematic review analyzed the evidence for potential disparities related to clozapine prescribing in psychotic disorders. The authors found that Black and Hispanic patients in the USA and UK were less likely to receive clozapine than non-Hispanic White patients. As clozapine is considered the gold standard for treatment-resistant psychosis, this points to a potential disparity in how schizophrenia spectrum disorders are managed.

### Inpatient and Emergency Psychiatric Services

*Hamilton JE, Heads AM, Cho RY, Lane SD, Soares JC. Racial Disparities During Admission to an Academic Psychiatric Hospital in a Large Urban Area. Compr Psychiatry. Nov 2015;63:113–22.* 10.1016/j.comppsych.2015.08.010.

The authors of this study retrospectively compared the care received by patients admitted to an urban, safety-net hospital. African American patients, as compared to non-Hispanic White patients, were more likely to receive a diagnosis of schizophrenia and were less likely to receive a diagnosis of an affective or anxiety disorder. This is inconsistent with population-based data demonstrating an equal prevalence of schizophrenia across these groups. The study results encourage psychiatrists to be mindful of diagnostic biases that may influence the care of ethnic and racial minority populations.

*Shea T, Dotson S, Tyree G, Ogbu-Nwobodo L, Beck S, Shtasel D. Racial and Ethnic Inequities in Inpatient Psychiatric Civil Commitment. Psychiatr Serv. Dec 1 2022;73(12):1322–1329.* 10.1176/appi.ps.202100342*.*

This prospective, single-site, cohort study followed individuals admitted to an inpatient unit to identify sociodemographic and clinical correlations to psychiatric commitment. Black and multiracial patients were more likely than White patients to have involuntary admission, even after adjusting for confounders. However, there were no significant differences in rates of filing a petition or proceeding to a court hearing based on race, possibly due to lack of power to detect a difference. This study highlights a concerning finding that Black and multiracial patients may receive inequitable exposure to involuntary admission.

*Eswaran V, Molina MF, Hwong AR, et al. Racial Disparities in Emergency Department Physical Restraint Use: A Systematic Review and Meta-analysis. JAMA Intern Med. Nov 1 2023;183(11):1229–1237.* 10.1001/jamainternmed.2023.4832

This systematic review and meta-analysis aimed to identify racial/ethnic disparities in the use of physical restraint in US emergency departments (EDs). Though use was observed in less than 1% of all encounters, Black patients were more likely to be restrained than were either White patients and all non-Black patients, raising concerns that Black patients are disproportionately restrained in US EDs. Psychiatrists should be mindful of this potential disparity when providing emergency care and considering restraint use.

### Outpatient

*Whiteley LB, Brown LK, Swenson R, Kapogiannis BG, Harper GW. Disparities in Mental Health Care Among HIV-Infected Youth. J Int Assoc Provid AIDS Care. Jan-Feb 2014;13(1):29–34.* 10.1177/2325957413488172

This cross-sectional survey of HIV infected adolescents and young adults (13–26 years old) enrolled at clinics specializing in HIV care examined the relationships between social and demographic variables and rates of psychiatric treatment. Of the respondents who self-reported qualifying mental health symptoms, Black youth were less likely than non-Black youth to have received mental health care and psychiatric medications. Latino patients were more likely than non-Latino patients to have received mental health care. HIV treatment sites and psychiatrists of HIV+ Black youth must continue to work to address this disparity.

*Heun-Johnson H, Menchine M, Axeen S, et al. Association Between Race/Ethnicity and Disparities in Health Care Use Before First-Episode Psychosis Among Privately Insured Young Patients. JAMA Psychiatry. Mar 1 2021;78(3):311–319.* 10.1001/jamapsychiatry.2020.3995

This cohort study of privately insured patients ages 10–21 examined potential disparities among patients with first-episode psychosis. The authors found that Black and Hispanic patients were less likely than White patients to have received a behavioral health diagnosis and treatment before first-episode psychosis was diagnosed. Black and Hispanic patients may have less opportunities for early detection of psychotic symptoms and early interventions. The authors propose potential interventions for clinicians to address this disparity, including increasing screening, practicing in underserved areas, and cultural competence.

*Pro G, Brown CC, Johnson O, Montgomery BEE, Zaller N. Comprehensive and Integrated Services in Specialty Mental Health Treatment Facilities in the US: Differences by the Racial/Ethnic Composition of the Facility’s Clientele, 2020. Community Ment Health J. Feb 2024;60(2):272–282.* 10.1007/s10597-023-01168-0

This cross-sectional study used the 2020 National Mental Health Services Survey to identify 12 different service types offered at mental health facilities and how the availability of these services varied based on the facility’s racial/ethnic composition. Facilities with the highest proportions of Black and Hispanic patients were least likely to offer comprehensive and integrated services. The authors suggest that disparities in mental health outcomes occur in part due to the structural inequities. This study supports the idea that mental health policy and funding should work to provide equitable distributions of comprehensive care.

### Inpatient Medical

*Charron E, Francis EC, Heavner-Sullivan SF, Truong KD. Disparities in Access to Mental Health Services Among Patients Hospitalized for Deliberate Drug Overdose. Psychiatr Serv. Sep 1 2019;70(9):758–764.* 10.1176/appi.ps.201800496

This retrospective study examined patient and hospital course characteristics associated with psychiatric assessment and inpatient psychiatric hospitalization following deliberate drug overdose. The authors found that non-Hispanic White patients were more likely to receive both mental health assessment and inpatient psychiatric admission following a deliberate drug overdose; minoritized races and ethnicities were less likely to receive either. These results point to a potential target for consultation-liaison and hospital-based psychiatrists to monitor for quality improvement.

*Garrett WS, Verma A, Thomas D, Appel JM, Mirza O. Racial Disparities in Psychiatric Decisional Capacity Consultations. Psychiatr Serv. Jan 1 2023;74(1):10–16.* 10.1176/appi.ps.202100685

This retrospective cross-sectional study aimed to assess racial and ethnic disparities in requests for consultation for decision making-capacity. Compared to the overall hospital population, Black patients were over-represented in consult requests. There were no differences in assessments of capacity based on race. This study indicates that there may be racial bias in requests for assessments of capacity and discusses how inappropriate requests for assessments of capacity may lead to psychological distress and increased distrust among Black individuals.

*Caravella RA, Ying P, Siegel C, et al. Quality Improvement Framework to Examine Health Care Disparities in Behavioral Emergency Management in the Inpatient Medical Setting: A Consultation-Liaison Psychiatry Health Equity Project. J Acad Consult Liaison Psychiatry. Jul-Aug 2023;64(4):322–331.* 10.1016/j.jaclp.2023.04.002

In this quality improvement study, the authors examined patient characteristics, including race and ethnicity, associated with behavioral emergency response interventions. Interventions included verbal de-escalation, parenteral medication administration, and restraints. Black patients were over-represented in the group receiving verbal de-escalation, Asian patients were over-represented in the group receiving parenteral medication, and patients identifying as Asian or Hispanic with a behavioral emergency alert were more likely to require interpreter services than other races/ethnicities. This study identified potential disparities in how behavioral alerts are managed, which psychiatrists, particularly hospital-based psychiatrists, should take into consideration when managing behavioral alerts.

### Addictions

*Pinedo M. Missed Opportunities by Health Care Providers to Reduce Racial/Ethnic Disparities in the Use of Alcohol Treatment Services. Drug Alcohol Depend. Sep 1 2021;226:108,851*. 10.1016/j.drugalcdep.2021.108851*.*

This nationally representative cross-sectional study investigated disparities in healthcare worker delivery of information regarding alcohol use disorder (AUD) treatment to racial and ethnic minority patients. In this study, Latino patients were less likely to be offered information about alcohol treatment than were African American and White patients. Among patients offered information about AUD treatment, there were no ethnic or racial differences in the use of treatment services. Such findings suggest that health care providers contribute to alcohol treatment disparities by missing a critical opportunity to encourage use of services among Latino patients.

*Mason M, Soliman R, Kim HS, Post LA. Disparities by Sex and Race and Ethnicity in Death Rates Due to Opioid Overdose Among Adults 55 Years or Older, 1999 to 2019. JAMA Netw Open. Jan 4 2022;5(1):e2142982.* 10.1001/jamanetworkopen.2021.42982

This cross-sectional study stratified the rates of death due to opioid overdose by adults 55 years or older from 1999 to 2019 by sex, race, and ethnicity. Overall rates of death due to opioid overdose increased significantly over this time, but by 2019, the fatality rate of non-Hispanic Black men in this study was four times greater than others of the same age. Psychiatrists, addiction specialists, and emergency clinicians must be diligent to provide screening, intervention, and treatment that helps to address this gap. Potential interventions include improving patient education and naloxone distribution to this population.

*Jackson DS, Nguemeni Tiako MJ, Jordan A. Disparities in Addiction Treatment: Learning from the Past to Forge an Equitable Future. Med Clin North Am. Jan 2022;106(1):29–41.* 10.1016/j.mcna.2021.08.008

This review provides a comprehensive history of the US drug policies that determine access to addiction treatment. The authors suggest that addiction treatment should be approached using a structurally competent framework that uses a diverse workforce to foster close collaboration between policymakers, health care systems, and clinicians. This work will require deliberate investment in training and supporting researchers and addiction specialists of color as well as investment in infrastructure and policy reform. Both cultural competency and advocacy are needed from psychiatrists to institute such change.

*Jones A, Santos-Lozada A, Perez-Brumer A, Latkin C, Shoptaw S, El-Bassel N. Age-Specific Disparities in Fatal Drug Overdoses Highest Among Older Black Adults and American Indian/Alaska Native Individuals of All Ages in the United States, 2015–2020. Int J Drug Policy. Apr 2023;114:103,977.* 10.1016/j.drugpo.2023.103977

This retrospective observational study assessed age-specific mortality rates in drug overdose deaths according to race and ethnicity. Overdose fatalities have impacted older Non-Hispanic Black adults and American Indian/Alaska Native populations at a greater incidence than Non-Hispanic White individuals. These findings underscore the need to focus substance use treatment and overdose prevention efforts on older Black adults and American Indian/Alaska Native individuals. Health care providers must continue to address structural racism and systematic discrimination as they implement culturally competent substance use treatment initiatives.

*Choi S, Hong S, Gatanaga OS, et al. Substance Use and Treatment Disparities Among Asian Americans, Native Hawaiians, and Pacific Islanders: A Systematic Review. Drug Alcohol Depend. Mar 1 2024;256:111,088.* 10.1016/j.drugalcdep.2024.111088

Despite rapidly increasing population size in the USA, Asian Americans, Native Hawaiian and Pacific Islander groups are underrepresented in substance use disorder (SUD) treatment facilities and research. This systematic review examined the prevalence of SUD in these populations, barriers to accessing treatment, and the efficacy of such treatment. The authors highlight the importance of disaggregating data to reveal distinct patterns within the ethnic subgroups that were mentioned, rather than including all under the category of “Asian.” Psychiatrists can champion research strategies to meet the needs of individual communities in relation to SUD treatment disparities.

### Child and Adolescent Psychiatry

*Alegria M, Vallas M, Pumariega AJ. Racial and Ethnic Disparities in Pediatric Mental Health. Child Adolesc Psychiatr Clin N Am. Oct 2010;19(4):759–74.* 10.1016/j.chc.2010.07.001

In this review, the authors explore mental health disparities in pediatrics, including the higher rates of mental health conditions in minority groups and the lower rates of mental health services received by minority groups. Several approaches to addressing disparities are discussed, including insurance expansion, community services, integrated services, and increasing cultural competence in healthcare. This paper can be used to provide an overview of disparities in child and adolescent psychiatry as well as provide an introduction to steps needed to combat them.

*Locke J, Kang-Yi CD, Pellecchia M, Marcus S, Hadley T, Mandell DS. Ethnic Disparities in School-Based Behavioral Health Service Use for Children with Psychiatric Disorders. J Sch Health. Jan 2017;87(1):47–54.* 10.1111/josh.12469

This retrospective analysis assessed behavioral health service use via Medicaid claims data in a large cohort of children within an urban school district in 2008–2009. In comparison with White children, Hispanic children had significantly lower use of in-school services and among children with attention-deficit/hyperactivity disorder (ADHD), African American children were less likely to receive in-school services. The article includes relevant policy information, discussion of potential factors including language barriers and cultural considerations, and implications for school health.

*Fadus MC, Ginsburg KR, Sobowale K, et al. Unconscious Bias and the Diagnosis of Disruptive Behavior Disorders and ADHD in African American and Hispanic Youth. Acad Psychiatry. Feb 2020;44(1):95–102.* 10.1007/s40596-019-01127-6

This narrative review summarizes evidence indicating that African American and Hispanic youth are more likely to receive a diagnosis of a disruptive behavior disorder and less likely to receive a diagnosis of ADHD compared to non-Hispanic White youth. The authors provide an evidence-based discussion of clinical and social impacts of these diagnoses and factors including unconscious bias, systemic/structural factors, and individual experiences likely involved in these diagnostic differences. The paper also discusses clear steps for individual providers and academic institutions to address these disparities.

*Foster AA, Porter JJ, Monuteaux MC, et al. Disparities in Pharmacologic Restraint Use in Pediatric Emergency Departments. Pediatrics. Jan 1 2023;151(1).* 10.1542/peds.2022-056667

In this retrospective cohort study, the authors examined utilization of pharmacologic restraints in pediatric (ages 3–21 years) patients over emergency department visits between 2010 and 2020. Patient characteristics associated with increased rates of pharmacological restraints included age of 18–21 years old, males, Black race, and holding public insurance. These findings indicate a potential disparity in utilization of pharmacological restraint in pediatric emergency departments for psychiatrists to be mindful of.

*Elliott TR, Choi KR, Elmore JG, Dudovitz R. Racial and Ethnic Disparities in Receipt of Pediatric Mental Health Care. Acad Pediatr. Aug 2024;24(6):987–994.* 10.1016/j.acap.2024.01.024

In this cross-sectional analysis, the authors examined correlations of receipt of mental health care in pediatric patients in the USA. The authors found that Hispanic and Black children were less likely to receive treatment than non-Hispanic White patients. Not having an established primary care physician (PCP) was also associated with decreased treatment rates. These data point to disproportionate access for minoritized children, as well as for children without a PCP, that psychiatrists should be mindful of.

*Pham AV, Charles LC. Racial Disparities in Autism Diagnosis, Assessment, and Intervention among Minoritized Youth: Sociocultural Issues, Factors, and Context. Curr Psychiatry Rep. May 2023;25(5):201–211.* 10.1007/s11920-023-01417-9

This review discusses structural inequities and sociocultural considerations in the diagnosis of autism spectrum disorder. The authors highlight that children belonging to minoritized groups experience delays in diagnosis of autism, experience barriers to interventions for autism treatment, and that assessments for autism spectrum disorder should take cultural considerations into account. Psychiatrists must be aware of these inequities and provide culturally competent care to address these disparities.

### Forensics

*Baglivio MT, Wolff KT, Piquero AR, Greenwald MA, Epps N. Racial/Ethnic Disproportionality in Psychiatric Diagnoses and Treatment in a Sample of Serious Juvenile Offenders. J Youth Adolesc. Jul 2017;46(7):1424–1451.* 10.1007/s10964-016-0573-4

This retrospective analysis utilized standardized assessment data from 2011 to 2014 for youth admitted to long-term juvenile justice resident placements in Florida. The authors found that, in comparison to White youth, Black youth were significantly more likely to be diagnosed with conduct disorder, Black and Hispanic males were less likely to be diagnosed with ADHD, and Black males were less likely to receive psychiatric treatment. Improved cultural competence and culturally sensitive diagnostic interviews may reduce these disparities.

*Wojciechowski TW. Racial Disparities in Community Mental Health Service Use Among Juvenile Offenders. J Racial Ethn Health Disparities. Apr 2019;6(2):393–400.* 10.1007/s40615-018-00536-x

This retrospective analysis utilized Pathways to Desistance data consisting of longitudinal self-reports of juvenile offenders (2000–2003). The authors found that Black and Hispanic youth utilize community mental health services at lower rates than White youth during the 6 months surrounding adjudication for a serious offense. The authors discuss potential system-level barriers impacting this disparate care utilization, one component of disparities within the youth justice system.

*Hobart KS, Krishnan S, Cleary SD, Candilis PJ. Assessing Racial Effects on Adjudicative Competence. J Am Acad Psychiatry Law. Dec 8 2023;51(4):542–550.* 10.29158/jaapl.230074-23

This retrospective cross-sectional study sought to evaluate differences in outcomes of evaluations for competency to stand trial for Black compared with White race in an urban, forensic setting. Measures included psychiatric diagnosis, prescription of antipsychotics, use of involuntary medication, use of restraint or seclusion, time to restoration of competence, and disposition back to jail. None of these measures differed significantly by race after adjusting for sociodemographic factors, but the authors did find that Black defendants were more likely to be referred for psychological testing than White defendants. Psychiatrists should be aware of potential process-level disparities as well as ensure equity in their decision-making.

### Geriatrics

*Cooper C, Tandy AR, Balamurali TB, Livingston G. A Systematic Review and Meta-analysis of Ethnic Differences in Use of Dementia Treatment, Care, and Research. Am J Geriatr Psychiatry. Mar 2010;18(3):193–203.* 10.1097/JGP.0b013e3181bf9caf

This systematic review aimed to compare the use of dementia services, treatment, and research between “minority ethnic” (ME) and non-ME groups. The authors found that ME groups had greater cognitive impairment at referral than non-ME patients and that Hispanic patients had greater duration of memory loss at referral. Compared with White patients, African-American (AA) individuals were less likely to be prescribed acetylcholinesterase inhibitors and were less likely to be represented in AD drug trials. While this study has limitations, such as vague inclusion criteria, this paper highlights important actionable opportunities to reduce disparities in research representation and medication prescription for ME patients with dementia.

*Kim G, Parton JM, DeCoster J, Bryant AN, Ford KL, Parmelee PA. Regional Variation of Racial Disparities in Mental Health Service Use Among Older Adults. Gerontologist. Aug 2013;53(4):618–626.* 10.1093/geront/gns107

This observational study examined racial and geographic differences in mental health service use among older adults. In this nationally representative sample, Black elders in the South were significantly less likely than White elders to use mental health services. The findings suggest that improving access to mental health care in certain regions, the South in particular, is essential to reducing racial disparities at a national level. The authors discuss policy implications of interest to psychiatrists in any geographical region.

*Pickett YR, Bazelais KN, Bruce ML. Late-Life Depression in Older African Americans: A Comprehensive Review of Epidemiological and Clinical Data. Int J Geriatr Psychiatry. Sep 2013;28(9):903–13.* 10.1002/gps.3908

This narrative review aimed to summarize the literature related to late-life depression in AA patients in the USA. Important findings include the replicated finding that, compared with White individuals, AA individuals are more likely to receive depression services in primary care, not psychiatry, clinics. In primary care clinics, depression in AA patients was less likely to be detected by primary care physicians, and AA individuals receive less depression treatment than White patients in primary care and nursing home settings. Psychiatrists must work to address this population’s needs.

*Cai S, Qin Q, Veazie P, Temkin-Greener H. Telemedicine and Disparities in Mental Health Service Use Among Community-Dwelling Older Adults with Alzheimer Disease and Related Dementias. J Am Med Dir Assoc. Jul 2024;25(7):105,027.* 10.1016/j.jamda.2024.105027

In this observational study, the authors examined rates of mental health care utilization in older adults with Alzheimer’s disease and related dementias (ADRD). The authors found that underserved racial and ethnic minorities with ADRD were less likely to receive mental health care. This gap improved, but persisted, with the telemedicine expansion during the pandemic. However, the gap for socioeconomically deprived communities worsened, highlighting that equitable care requires equitable access to technology in addition to mental health care services.

### Neuromodulation

*Luccarelli J, Henry ME, McCoy TH, Jr. Demographics of Patients Receiving Electroconvulsive Therapy Based on State-Mandated Reporting Data. J ect. Dec 2020;36(4):229–233.* 10.1097/yct.0000000000000692

In this observational study, the authors found a disproportionate administration of electroconvulsive therapy (ECT) to women, patients over the age of 65, and White patients. As ECT has been shown to be an effective treatment for depression, catatonia, and other psychiatric disorders, psychiatrists should be mindful of these disparities in order to provide equitable care and referrals to ECT services.
